# Acute Kidney Injury in Patients with Newly Diagnosed High-Grade Hematological Malignancies: Impact on Remission and Survival

**DOI:** 10.1371/journal.pone.0055870

**Published:** 2013-02-14

**Authors:** Emmanuel Canet, Lara Zafrani, Jerome Lambert, Catherine Thieblemont, Lionel Galicier, David Schnell, Emmanuel Raffoux, Etienne Lengline, Sylvie Chevret, Michael Darmon, Elie Azoulay

**Affiliations:** 1 Medical Intensive Care Unit, Saint-Louis University Hospital, Paris, France; 2 Biostatistics Department, Saint-Louis University Hospital, Paris, France; 3 Department of Hematology and Oncology, Saint-Louis University Hospital, Paris, France; 4 Department of Clinical Immunology, Saint-Louis University Hospital, Paris, France; 5 Department of hematology, Saint-Louis University Hospital, Paris, France; 6 Medical-Surgical Intensive Care Unit, Saint-Etienne University Hospital, Saint-Etienne, France; 7 Université Paris Diderot, Sorbonne Paris Cité, Paris, France; Robert Wood Johnson Medical School, United States of America

## Abstract

**Background:**

Optimal chemotherapy with minimal toxicity is the main determinant of complete remission in patients with newly diagnosed hematological malignancies. Acute organ dysfunctions may impair the patient’s ability to receive optimal chemotherapy.

**Design and Methods:**

To compare 6-month complete remission rates in patients with and without acute kidney injury (AKI), we collected prospective data on 200 patients with newly diagnosed high-grade malignancies (non-Hodgkin lymphoma, 53.5%; acute myeloid leukemia, 29%; acute lymphoblastic leukemia, 11.5%; and Hodgkin disease, 6%).

**Results:**

According to RIFLE criteria, 137 (68.5%) patients had AKI. Five causes of AKI accounted for 91.4% of cases: hypoperfusion, tumor lysis syndrome, tubular necrosis, nephrotoxic agents, and hemophagocytic lymphohistiocytosis. Half of the AKI patients received renal replacement therapy and 14.6% received suboptimal chemotherapy. AKI was associated with a lower 6-month complete remission rate (39.4% vs. 68.3%, *P*<0.01) and a higher mortality rate (47.4% vs. 30.2%, *P*<0.01) than patients without AKI. By multivariate analysis, independent determinants of 6-month complete remission were older age, poor performance status, number of organ dysfunctions, and AKI.

**Conclusion:**

AKI is common in patients with newly diagnosed high-grade malignancies and is associated with lower complete remission rates and higher mortality.

## Introduction

Hematologic malignancies cause many deaths worldwide and their incidence is increasing [1]. Over the past 20 years, important insights have been gained into the pathophysiology of hematologic malignancies, intensified treatments have been developed, and new targeted treatments have been introduced. Novel strategies include a monoclonal antibody against tumor cells for non-Hodgkin lymphoma (NHL) [2], intensive chemotherapy regimens for Ph-positive acute lymphoblastic leukemia (ALL) [3] and Burkitt lymphoma [4], and combination chemotherapy comprising all-trans retinoic acid for acute promyelocytic leukemia [5]. These advances have provided high remission rates and improved survival. However, intensive chemotherapy has increased the occurrence of life-threatening toxic and infectious complications, thus increasing the need for intensive care unit (ICU) admission [6].

Acute kidney injury (AKI) is a dreaded complication in patients with hematological malignancies that may be caused by the disease itself or by its treatment [7]. Of the many factors that can cause AKI in this setting, sepsis and hypoperfusion have been reported to be the most common [8], [9]. Other causes include metabolic complications (tumor lysis syndrome [TLS] and hypercalcemia), renal infiltration by neoplastic cells, obstructive nephropathy, glomerulonephritis, and treatment-induced nephrotoxicity. AKI markedly increases the duration of hospitalization and the cost of care in unselected hospitalized patients. Also, even small changes in serum creatinine levels are associated with increased mortality [10]. Among overall critically ill patients, AKI-related mortality rates exceed 30% and reach 60% if renal replacement therapy is required [11]. AKI can also preclude optimal management of the malignancy, as changes in chemotherapeutic drug pharmacokinetics related to renal dysfunction may result in underdosing, with a decreased chance of achieving a remission, or in overdosing, with an increased risk of toxicity. The exact incidence of AKI in patients with hematological malignancies has been difficult to ascertain in the past given the absence of a universally accepted definition. The RIFLE classification scheme for AKI developed in 2004 is highly sensitive and clinically relevant and has been validated in numerous studies involving over 200,000 patients [11], [12], [13]. RIFLE distinguishes three severity categories (risk, injury, and failure) based on glomerular filtration rate or urine output reduction. Patients meeting RIFLE criteria have poorer outcomes, even after adjustment for co-variates likely to affect mortality. Few data are available on the impact of AKI on outcomes of patients with hematological malignancies since the introduction of novel treatment strategies.

The goal of this study was to assess whether early AKI decreased the complete remission (CR) rate in patients with newly diagnosed high-grade hematological malignancies. To this end, we conducted a hospital-based prospective observational cohort study. We also determined the incidence, clinical features, and mortality associated with AKI in this population.

## Materials and Methods

This prospective observational study was approved by the Ethics committee from the institutional review board of the Bichat university hospital (CEERB) in Paris, France. Patients or surrogate decision makers were given a specific form explaining the study objectives, the obervational design and the anonymous data collection. According to the French law and to our IRB recommandations, written consent was not required for this non interventional study. Patients or surrogates provided verbal informed consent prior to study inclusion. Verbal consent was obtained through a session of patient or family information explaining the study, its aims and the non interventional design. The consent was recorded in the medical chart of each patient. Patients or relatives had the opportunity to decline study participation at any time. No data allowing patient identification were collected.

### Design and Setting

We included consecutive adults admitted to our ICU in the Saint-Louis University Hospital, Paris, France, between November 1, 2007, and October 31, 2010, with newly diagnosed high-grade hematological malignancies that were either not yet treated or being treated with first-line chemotherapy. Patients were admitted to the ICU either from outside the hospital or from one of the seven hematology wards in the hospital. The Saint-Louis University Hospital is a 650-bed public hospital with 330 beds for patients with hematologic malignancies and solid cancers. The ICU is a 12-bed medical unit that admits 750–850 patients per year, of whom about one-third have hematological malignancies. Information on the organization of our ICU and criteria for ICU admission have been published elsewhere [14]. ICU admission policies remained unchanged throughout the study period.

The following malignancies were considered in this study: acute myelogenous and lymphoblastic leukemias, high-grade NHL, and Hodgkin disease [15]. Exclusion criteria were prior chronic renal dysfunction, previously treated malignancy (>4 weeks after the first chemotherapy dose), relapsing or refractory hematological malignancy, bone marrow or stem cell transplantation, low-grade or chronic malignancy (chronic myelogenous leukemia, chronic lymphocytic leukemia, low-grade NHL, multiple myeloma, myelodysplastic syndrome, myelofibrosis with myeloid metaplasia, or aplastic anemia), and absence of potentially lifespan-extending treatment options.

At our institution, senior hematologists and intensivists are available 24 hours a day 7 days a week and work together to manage all high-risk hematology patients. AKI was diagnosed when the patients met RIFLE criteria [12]. Decisions regarding the initiation, discontinuation, and modalities of renal replacement therapy (RRT) were taken by senior nephrologists (EC, LZ, and MD), based on the guidelines from Ronco and Bellomo [16]. Chemotherapy was prescribed by the hematologist in charge of the patient, according to best standard of care. After ICU discharge, all patients were managed in our hospital, and 6-month follow-up data were available for all of them. Patients were classified as alive with CR, alive with refractory disease or relapse, or dead. Assessment of CR was achieved according to recent criteria for each hematological malignancy [17], [18], [19].

### Data Collection

The data in Tables 1, 2, 3 and S1, S2 were collected prospectively. AKI stage was assessed based on the worst RIFLE class within 7 days after ICU admission. The clinical, laboratory, and imaging data in each patient were reviewed by the intensivist/nephrologists, who reached a consensus regarding the diagnosis of AKI. Great care was taken to identify all exposures to nephrotoxic agents (radiographic contrast media, vancomycin, aminoglycosides, and nonsteroidal antiinflammatory drugs). All patients underwent urine cultures and either renal ultrasound or non-contrast-enhanced computed tomography. Causes of AKI were hypoperfusion, acute tubular necrosis, TLS, kidney infiltration by malignant cells, urinary tract obstruction, hemolytic-uremic syndrome, hemophagocytic lymphohistiocytosis, pyelonephritis, and drug nephrotoxicity. The Sequential Organ Failure Assessment (SOFA) score was computed based on the highest score within 7 days after ICU admission [20]. This score has been developed in unselected critical ill patients to correlate the number and severity of organ dysfunctions with hospital mortality. Life-sustaining therapies (oxygen, noninvasive mechanical ventilation, mechanical ventilation, vasopressors, and RRT) were used according to the best standard of care.

**Table 1:** Patient characteristics (n = 200) at ICU admission.

Variables N (%) or median [IQR]		All patients (n = 200)	AKI (n = 137)	No AKI (n = 63)	*P* value
**Demographics**					
Age (yr)		51 [35;62]	51 [40;63]	48 [30;59]	0.28
Male gender,		120 (60)	87 (63.5)	33 (52.4)	0.16
Co-morbidities,					
*Hypertension*		41 (20.5)	32 (23.4)	9 (14.3)	0.19
*Heart failure*		31 (15.5)	20 (14.6)	11 (17.5)	0.68
*Diabetes mellitus*		17 (8.5)	11 (8)	6 (9.5)	0.79
*HIV infection*		42 (21)	30 (21.9)	12 (19)	0.71
**ECOG Performance status**					0.20
0–2		129 (64.5)	84 (61.3)	45 (71.4)	
3–4		71 (35.5)	53 (38.7)	18 (28.6)	
**Type of hematological malignancy, n (%)**					0.94
*Non-Hodgkin lymphoma*		107 (53.5)	72 (52.6)	35 (55.6)	
*Acute myeloid leukemia*		58 (29)	40 (29.2)	18 (28.6)	
*Acute lymphoblastic leukemia*		23 (11.5)	17 (12.4)	6 (9.5)	
*Hodgkin disease*		12 (6)	8 (5.8)	4 (6.3)	
Time (days) since diagnosis,		3 [0;24]	2 [0;18]	4 [0;32]	0.09
Ongoing chemotherapy, n (%)		192 (96)	135 (98.5)	56 (88.8)	<0.01
Time (days) since chemotherapy		1 [0;14.5]	1 [0;11.8]	2 [0;25]	0.42
**Exposure to nephrotoxic drugs**		140 (70)	99 (72.3)	41 (65.1)	0.32
**Laboratory data at ICU admission**					
Leukocyte count (G/L)		8.05 [1.55;24.05]	9.5 [2.0;32.6]	6.1 [0.95;13.7]	0.05
Neutropenia (≤1.0G/L), n (%)		45 (22,5)	28 (20.4)	17 (27)	0.47
DIC, n (%)		51 (25.5)	42 (30.7)	9 (14.3)	0.01
Serum creatinine (µmol/L)		84 [66;135]	103 [76;164]	66 [50;78]	<0.01
Uric acid (µmol/L),		309 [159;540]	401 [197;615]	189 [96;348]	<0.01
Lactate dehydrogenase (Units/L),		1314 [670;2777]	1698 [852;3541]	802 [466;1468]	<0.01
**Reason for ICU admission, n (%)**					<0.01
Close monitoring		32 (16)	13 (9.5)	19 (30.2)	
Acute respiratory failure		48 (24)	28 (20.4)	20 (31.7)	
Severe sepsis - Septic shock		49 (24.5)	36 (26.3)	13 (20.6)	
Neurologic disorder		19 (9.5)	9 (6.6)	10 (15.9)	
Metabolic disturbances		52 (26)	51 (37.2)	1 (1.6)	
**SOFA**		7 [4]; [11]	8 [6]; [13]	5 [2]; [7]	<0.01
**SOFA** (Renal SOFA excluded),		6 [3]; [10]	6 [4]; [10]	5 [2]; [7]	<0.01
**ICU management, n (%)**					
Non-invasive mechanical ventilation		27 (13.5)	20 (14.6)	7 (11.1)	0.66
Mechanical ventilation		82 (41)	64 (46.7)	18 (28.6)	0.02
Vasopressors		84 (42)	69 (50.4)	15 (23.8)	<0.01
Renal replacement therapy		72 (36)	72 (52.6)	0 (0)	<0.01
Chemotherapy		134 (67)	94 (68.6)	40 (63.5)	0.52

ICU, intensive care unit; AKI, acute kidney injury; DIC, disseminated intravascular coagulopathy; RRT, renal replacement therapy. SOFA : Sequential Organ Failure Assessment. This score has been developed in unselected critical ill patients to correlate the number and severity of organ dysfunctions with hospital mortality. We report the highest score within 7 days after ICU admission.

**Table 2:** Characteristics of acute kidney injury (n = 137).

Variables	n (%) or median [IQR]
**RIFLE classification within 7 days after ICU admission**	
*Risk*	37 (27)
Serum creatinine (µmol/L)	79 [65;98]
*Injury*	27 (19.7)
Serum creatinine (µmol/L)	117 [85;138]
*Failure*	73 (53.3)
Serum creatinine (µmol/L)	143 [86;244]
Oliguria	77 (56.2)
**Laboratory data**	
Proteinuria (g/L)	0.47 [0.22;0.92]
Hematuria	40 (31.5)
Serum albumin (g/L)	28 [23]; [33]
Positive urine culture	19 (15.1)
**Exposure to nephrotoxic drugs**	99 (72.3)
Radiographic contrast agents	33 (24.1)
Vancomycin	60 (43.8)
Aminoglycoside	77 (56.2)
Nonsteroidal antiinflammatory drugs	9 (6.6)
**Causes of AKI**	
Hypoperfusion	66 (48.2)
Tumor lysis syndrome	60 (43.8)
Acute tubular necrosis	39 (28.5)
Nephrotoxic agents	28 (20.4)
Hemophagocytic lymphohistiocytosis	20 (14.6)
Kidney infiltration by malignancy	8 (5.8)
Urinary tract obstruction	6 (4.4)
Pyelonephritis	5 (3.7)
Hemolytic-uremic syndrome	1 (0.7)
More than one cause of AKI	62 (45.3)
**Renal replacement therapy during ICU stay**	72 (52.6)
**Modification of chemotherapy because of AKI**	20 (14.6)
Dose reduction	
* Cytarabine*	4 (20)
* Cyclophosphamide*	3 (15)
* Oxaliplatin*	1 (5)
Withdrawal	
* Methotrexate*	10 (50)
* Cytarabine*	2 (10)
**Follow-up data on renal function**	
RRT at ICU discharge	28 (20.4)
Serum creatinine (µmol/L) at ICU discharge in RRT-free patients	66 [52;102]
Serum creatinine (µmol/L) at month 3	62 [52;75]
Serum creatinine (µmol/L) at month 6	67 [52; 79]
Renal recovery among hospital survivors (n = 89)	82 (92.1)

ICU, intensive care unit; AKI, acute kidney injury; RRT, renal replacement therapy.

**Table 3:** Outcome data.

Variables Median [IQR] or n (%)	All patients (n = 200)	AKI (n = 137)	No AKI (n = 63)	*P* value
**Outcome data**				
ICU length of stay	7 [4]; [14]	8 [3]; [17]	4 [2]; [6]	<0.01
Hospital length of stay	43 [33;79]	45 [33;79]	40 [28;68]	0.74
ICU mortality	38 (19)	33 (24)	5 (7.9)	<0.01
Hospital mortality	60 (30)	48 (35)	12 (19.1)	0.02
**Hematological status at month 6**				
Alive with persistent complete remission	97 (48.5)	54 (39.4)	43 (68.3)	<0.01
Alive with relapse or refractory disease	18 (9)	17 (12.4)	1 (1.6)	
Death	84 (42)	65 (47.4)	19 (30.2)	
Lost to follow-up	1 (0.5)	1 (0.7)	0 (0)	

IQR, interquartile range; AKI, acute kidney injury.

In each patient with AKI, hematologist consultants determined whether chemotherapy was suboptimal because AKI contraindicated the use of a full-dose regimen and/or the use of one or more classes of chemotherapy agents.

Vital status 6 months after ICU discharge was available for all patients but one who was lost to follow-up after hospital discharge. In addition, hematological status (alive with complete remission, alive with relapsing or refractory malignancy, or dead), and serum creatinine at ICU discharge and 3 and 6 months after ICU discharge were available.

### Statistical Analysis

Data are presented with median [25^th^–75^th^ percentile] for continuous variable and frequency (percentage) for qualitative variables. Acute kidney injury (AKI) was defined using the most severe RIFLE class within 7 days after ICU admission. Characteristics of patients with and without AKI were compared using either the Wilcoxon sum-rank test or the Fisher’s exact test when appropriate. The primary endpoint was complete remission (CR) of the hematological malignancy at 6 months after ICU admission. Characteristics of patients with and without CR were compared using logistic regression, and variables associated with complete remission in the univariate analysis at a p = 0.05 level were included in a multivariate model with stepwise backward and forward selection. Results are presented as odds ratio and their 95% confidence interval. At each time, a patient can either be alive with the hematological malignancy (which encompasses newly diagnosed malignancy, refractory disease and relapse after complete remission), alive in CR, or dead. Multi-state modeling allows assessing the transition probabilities between these 3 states during the follow-up. The overall survival and the current malignancy-free survival, defined as the probability that a patient is alive and in CR, are assessed. Thus at each time of the follow-up after ICU discharge the proportion of patients alive in CR, proportion of patients alive with hematological malignancy and proportion of patients deceased are displayed on the same figure. This analysis was done for the whole cohort and stratified according to presence of AKI. All tests were two sided at p = 0.05 level and analysis were performed using R software version 2.14 [21].

## Results

### Study Population

**Figure 1:**
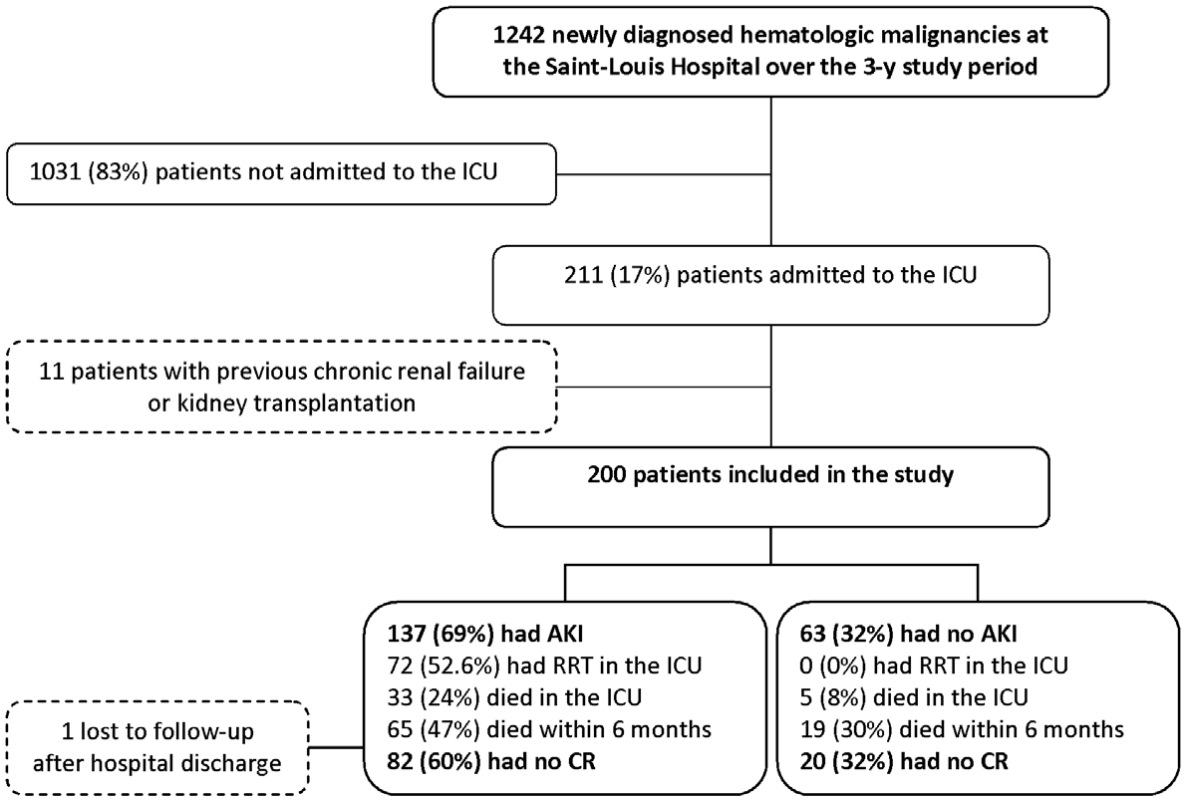
Flow-chart of the 200 patients with high-grade newly diagnosed hematological malignancies included in the study (53.5% non-Hodgkin lymphoma, 29% acute myeloid leukemia, 11.5% acute lymphoblastic leukemia, 6% Hodgkin disease). AKI, acute kidney injury; ICU, intensive care unit; RRT, renal replacement therapy; CR, complete remission. AKI was defined based on RIFLE criteria.

Among the 1242 patients with newly diagnosed high-grade hematological malignancies at the Saint-Louis University Hospital during the study period, 211 (17%) were admitted to our ICU and 200 were included. Follow-up data were available at 6 months for all patients but one, who was lost to follow-up after hospital discharge (Figure 1). Patient characteristics are reported in Table 1. Most common malignancies were non-Hodgkin lymphoma, acute myeloid leukemia and acute lymphoblastic leukemia. Median times to ICU admission were 3 days (IQR, 0 to 24) after malignancy diagnosis and 1 day (IQR, 0 to 14) after chemotherapy initiation.

### Characteristics of Acute Kidney Injury

Of the 200 patients, 137 (68.5%) fulfilled AKI criteria, of whom 27% were in the Risk category, 19.7% in the Injury category, and 53.3% in the Failure category (Table 2). Among AKI patients, 72.3% had prior exposure to nephrotoxic drugs. Five causes of AKI accounted for 91.4% of cases: hypoperfusion, TLS, acute tubular necrosis, nephrotoxic agents and hemophagocytic lymphohistiocytosis.

Renal replacement therapy was used in 72 patients (52.6%) throughout the ICU stay. The modalities of renal replacement therapy were intermittent hemodialysis (63.8%), continuous hemofiltration (19.4%), or alternative use of both techniques (16.6%). Twenty-eight patients (20.4%) were RRT-dependent at ICU discharge with a hospital mortality rate of 85.7%.

Due to AKI, the chemotherapy regimen was subsequently modified in 20 patients (14.6%). Modifications consisted of reduced dosing in 8 cases and chemotherapy withdrawal in 12 cases. The drugs involved were either drugs with known nephrotoxicity, and/or drugs requiring renal clearance: methotrexate, aracytine, cyclophosphamide and oxaliplatin.

### Outcome Analysis

**Figure 2:**
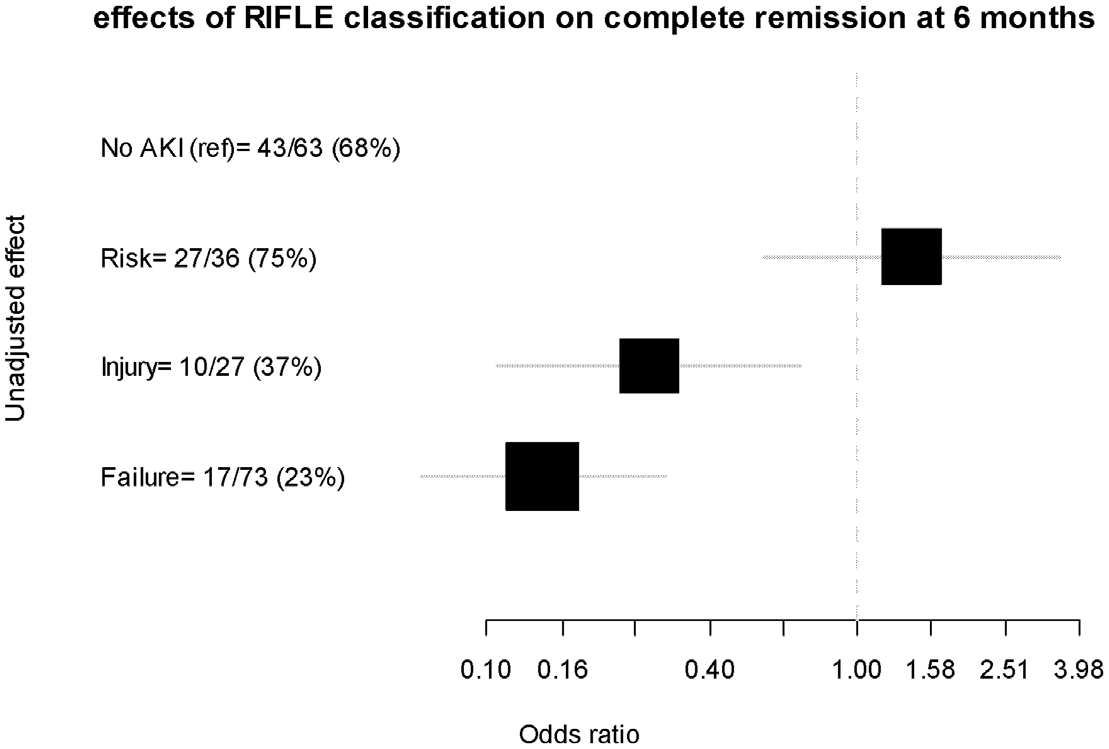
Effects of RIFLE classification on complete remission at 6 months. RIFLE classification scheme for acute kidney injury (AKI). The classification system includes separate criteria for creatinine and urine output (UO). A patient can fulfill the criteria through changes in serum creatinine (SCreat) or changes in UO, or both within 7 days. The criteria that lead to the worst possible classification should be used. R, RIFLE risk category: increased SCreat×1.5 or Glomerular filtration Rate (GFR) decrease >25% or UO <0.5 ml/kg/h×6 hours. I, RIFLE injury category: increased SCreat×2 or GFR decrease >50% or UO <0.5 ml/kg/h×12 hours. F, RIFLE failure category: increased SCreat×3 or SCreat greater than 4.0 mg/dl (350 µmol/l) with an acute increase of at least 0.5 mg/dl (44 µmol/l) or GFR decrease >75% or UO <0.3 ml/kg/h×24 hours or anuria×12 hours.

AKI was associated with a significantly lower 6-month CR rate (39.4% vs. 68.3%, *P*<0.01) (Table 3).The probability of being in CR decreased with AKI according to the RIFLE classification, whether RRT was required or not (Figure 2).

**Figure 3:**
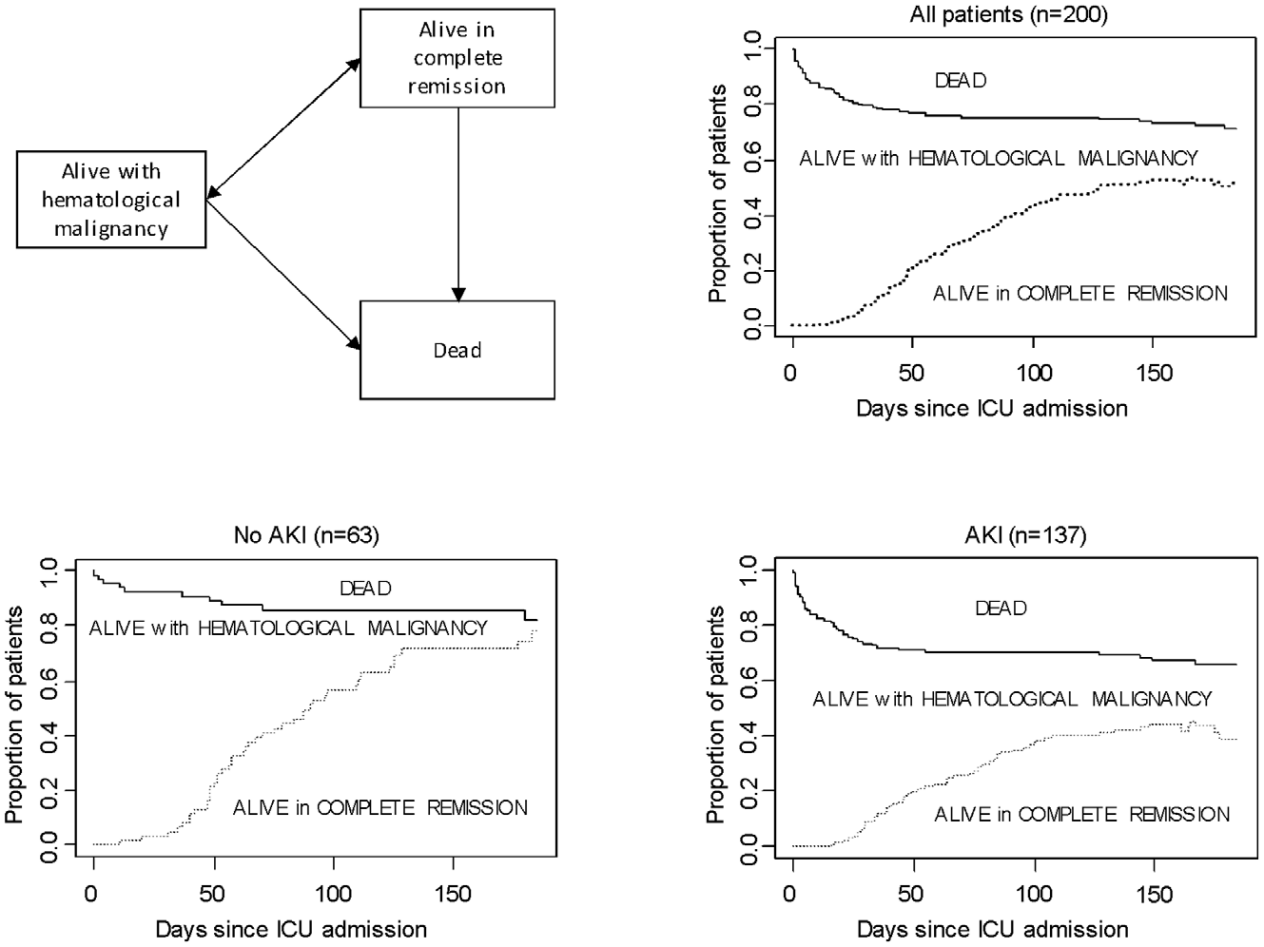
6-month outcome probabilities in patients with newly diagnosed hematological malignancies. A three-state model was used: alive with hematological malignancy, alive in complete remission, and dead. All patients start in state alive with hematological malignancy. The black line represents the overall survival and the dashed line current survival free of malignancy, defined as the probability of being alive and in complete remission. Results are presented for the whole cohort and stratified according to the presence of AKI at day 1. AKI, acute kidney injury; ICU, intensive care unit.

Figure 3 shows the overall survival and the current malignancy-free survival for the whole cohort and stratified according to the presence of AKI after ICU discharge. At each time of the follow-up, patients with AKI had higher rates of relapse, refractory disease and mortality.

Table 4 lists the variables independently associated with 6-month CR: older age, poor performance status, SOFA score, and AKI. Interestingly, beside poor performance status, AKI also predicted a lower 6-month complete remission rate in the subgroup of 162 ICU survivors (Table 5). This result suggests that the impact of AKI on CR was not only explained by an increase of short-term mortality.

**Table 4:** Multivariate analysis: predictors of complete remission of the hematological malignancy at 6 months.

	No. patients with CR /total No. patients (%)	Odds ratio	95% confidence interval	*P* value
**Age (yr), per 5-year increments**		0.89	[0.79;1.00]	0.04
**Performance Status**				<0.01
0–1–2	77/129 (60%)	1	–	
3–4	20/71 (28%)	0.26	[0.13;0.55]	
**SOFA (Renal SOFA excluded)**		0.87	[0.80;0.94]	<0.01
**RIFLE classification of AKI**				<0.01
No AKI	43/63 (68%)	1	–	
Risk	27/37 (75%)	1.36	[0.49;3.77]	
Injury	10/27 (37%)	0.31	[0.11;0.88]	
Failure	17/73 (23%)	0.22	[0.09;0.50]	

Candidate predictors were age, reason for ICU admission, HIV infection, SOFA (Renal SOFA excluded), RIFLE classification of AKI and Performance Status. ICU, intensive care unit; HIV, human immunodeficiency virus; SOFA, Sequential Organ Failure Assessment; AKI, acute kidney injury. For SOFA and RIFLE, the highest score within 7 days after ICU admission were considered. le Cessie - van Houwelingen goodness of fit test: p-value = 0.54, C-statistic = 0.83.

**Table 5:** Multivariate analysis: predictors of complete remission of the hematological malignancy at 6 months among the 162 ICU survivors.

	No. patients with CR/total No. patients (%)	Odds ratio	95% confidence interval	*P* value
**Performance Status**				<0.01
0–1–2	77/107 (72%)	1	–	
3–4	20/54 (37%)	0.26	[0.12;0.54]	
**RIFLE classification of AKI**				<0.01
No AKI	43/58 (74%)	1	–	
Risk	27/35 (77%)	1.19	[0.43;3.32]	
Injury	10/25 (40%)	0.23	[0.08;0.66]	
Failure	17/43 (40%)	0.27	[0.11;0.67]	

Candidate predictors were age, reason for ICU admission, HIV infection, SOFA (Renal SOFA excluded), RIFLE classification of AKI and Performance Status. ICU, intensive care unit; HIV, human immunodeficiency virus; SOFA, Sequential Organ Failure Assessment; AKI, acute kidney injury. For SOFA and RIFLE, the highest score within 7 days after ICU admission were considered. le Cessie - van Houwelingen goodness of fit test: p-value = 0.90, C-statistic = 0.75.

The etiology of AKI was also significantly associated with the 6-month CR rate. Six-month CR rates in patients with AKI due to TLS were not significantly different from those in patients without AKI. All other causes of AKI were associated with lower CR rates compared to patients without AKI (Table S1). However, the etiology was no longer significantly associated with CR after adjustment on AKI severity (data not shown).

## Discussion

This is the largest prospective study assessing outcomes in patients with newly diagnosed high-grade hematological malignancies according to presence or absence of AKI. Seventeen percent of all patients with hematological malignancies newly diagnosed at our hospital required ICU admission. Among the patients admitted to the ICU, 81% survived to ICU discharge and almost half were in complete remission after 6 months. Based on RIFLE criteria, AKI was present in 68.5% of the ICU patients. AKI required dialysis in 36% of cases and prevented optimal chemotherapy in 15%. AKI reduced the probability of achieving complete remission at 6 months. Of the patients who were dependent on dialysis at ICU discharge, 85% died within 6 months.

We used a standardized definition of AKI, in recognition of the potential clinical importance of small changes in kidney function [10]. The RIFLE criteria provide a graded definition of AKI severity [12]. Previous studies may have underestimated the occurrence of AKI due to the low sensitivity of other markers of renal insufficiency. In keeping with this possibility, the rate of AKI in our study was twice as high as in previously published cohort studies of unselected ICU patients [11] or cancer patients admitted to the ICU [8], [22]. Another possible explanation to this discrepancy is that 96% of our patients started to receive cancer chemotherapy 1 day (0–14) before ICU admission. Cancer chemotherapy often causes AKI, via several mechanisms such as dehydration due to gastrointestinal toxicity, TLS, and direct nephrotoxicity. Furthermore, 22.5% of our patients had neutropenia and were therefore at increased risk for severe sepsis, a major factor contributing to cause AKI in critically ill patients. In addition, a large proportion of our patients (70%) had been exposed to nephrotoxic drugs. However, as reported in other studies, renal recovery occurred in more than 90% of ICU survivors [23].

Our patients were managed jointly by intensivists, nephrologists, and hematologists. This multidisciplinary approach allowed us to determine the etiologies of AKI. In agreement with earlier works, the main causes of AKI were sepsis (with renal hypoperfusion) and acute tubular necrosis [8], [9], [24]. The next more common etiologies were toxicity and TLS, which contributed to the development of AKI in 53.3% of our patients. In an earlier study of cancer patients, the leading factors associated with AKI were nephrotoxic agents, TLS, and disseminated intravascular coagulopathy [9]. Similarly, in our study, a high tumor burden (as reflected by serum levels of lactic dehydrogenase and uric acid) was associated with higher rates of AKI (Table S2). Early identification of the etiologies of AKI is crucial to ensure appropriate treatment, such as adequate hydration, urate oxidase therapy to prevent uric acid-related nephropathy, and RRT to prevent acute nephrocalcinosis [25]. Hemophagocytic lymphohistiocytosis contributed to the development of AKI in 14.6% of our patients, in keeping with previous data [26]. Renal manifestations of hemophagocytic lymphohistiocytosis include tubular necrosis, interstitial nephritis, collapsing glomerulopathy, and minimal-change glomerulopathy [27], [28]. Patients fulfilling HLH-2004 criteria may require early specific therapy [29], [30].

A major finding from the present study is that AKI in patients with newly diagnosed high-grade hematological malignancies reduced the likelihood of achieving CR. This effect of AKI was also found in the subgroup of 162 ICU survivors. It means that this finding was not only explained by the increased short-term mortality in AKI patients. To the best of our knowledge, this effect of AKI has not been reported previously. Earlier works focused on short- and long-term mortality to assess the impact of AKI in patients with hematological malignancies [8], [15], [31], [32]. All studies consistently showed higher mortality rates in the patients with AKI compared to those without AKI. We found that AKI was associated not only with mortality, but also with failure of survivors to achieve a complete remission. This effect was evident even in patients who received full chemotherapy (85.4%) and in those who did not require RRT. Thus, AKI seems to decrease the chances of chemotherapy being optimally effective, even when the full regimen is given. One possible explanation may pertain to the lack of knowledge on drug pharmacokinetics in patients with renal dysfunction. Also, AKI may exert deleterious systemic effects that jeopardize the effectiveness of chemotherapy. Finally, AKI may be a marker of adverse prognostic significance in patients with hematological malignancies. Not surprisingly, the administration of suboptimal chemotherapy because of AKI was associated with a very poor prognosis in our study, with only 25% of patients being alive in complete remission at 6 months. Methotrexate is among the most problematic drugs in patients with AKI, because it is cleared predominantly by the kidneys and may therefore need to be given in reduced dosages or withdrawn in patients with AKI. High-dose methotrexate is part of the treatment regimen for acute lymphoid leukemia and high-grade lymphoma [33], [34], [35], [36], [37]. In Burkitt lymphoma, less intensive regimens without methotrexate are associated with high relapse rates [38], [39]. In our study, of the 10 patients who were unable to receive methotrexate because of AKI, only 1 was alive and in complete remission at 6 months. Beside AKI, 2 patients with liver insufficiency and 1 patient with cardiac insufficiency received suboptimal chemotherapy. However these patients were all with poor performance status, a variable strongly associated with the ability to achieve CR at 6 months. Studies assessing the impact of liver and cardiac insufficiencies on the outcome of patients with newly diagnosed malignancies are warranted.

Importantly, our study shows that the etiology influences the prognostic significance of AKI. Patients with AKI due only to TLS had the same likelihood of being in complete remission after 6 months as did patients without AKI. This finding is not surprising, as TLS is a marker for a good tumor response to chemotherapy. However, when AKI severity was taken into account, the cause of AKI no longer significantly affected its prognostic impact. Along this line, early recognition and management of high-risk patients for clinical TLS might improve outcome (Table S1).

Our study has several limitations. First, it was conducted at a single institution. Our admission policy and patient recruitment patterns may have influenced the findings. However, the large number of hematological patients treated in the numerous hematology wards at our hospital and the standardized policy of early ICU admission [14], [40] (as indicated by the fact that 16% of patients were admitted for close monitoring) suggest that our results may also apply to other settings. Second, renal biopsies were not performed, either because of a high risk of bleeding or because the renal disorder was not severe. Therefore, we may have underestimated the incidence of difficult-to-diagnose causes of AKI such as kidney infiltration by the malignancy. Third, as various high-grade malignancies were included in this study, we were unable to individualize prognostic factors of each malignancy. Thus, it could not be excluded that AKI was a surrogate marker of poor prognosis malignancies. The strengths of our study include the large and homogeneous patient population recruited over a short period (3 years) during which no changes in treatment practices occurred. Also, our unit has extensive experience in managing medical complications of hematological malignancies [14], [29], [41], [42], [43].

In summary, AKI is common in patients with newly diagnosed high-grade hematological malignancies. Similar proportions of patients with acute leukemia and high-grade lymphoma develop AKI. AKI decreases the chances of achieving a complete remission and adversely affects survival. Early intensive management including nephrotoxic drug withdrawal, adequate assessment and prevention of TLS, and early administration of specific treatments might improve patient outcomes when combined with aggressive supportive care.

## Supporting Information

Table S1 Influence of the cause of acute kidney injury on the 6-month complete remission rate. CR, complete remission; TLS, tumor lysis syndrome; AKI, acute kidney injury.
(DOC)

Table S2 Multivariate analysis: predictors of AKI.
(DOC)
