# Supplementary material for: Acute Kidney Injury in Patients with Newly Diagnosed High-Grade Hematological Malignancies: Impact on Remission and Survival
Source: PLoS One. 2013 Feb 14;8(2):e55870. doi: 10.1371/journal.pone.0055870 (PMC3573047; doi:10.1371/journal.pone.0055870)
Supplement: Table S1 — Influence of the cause of acute kidney injury on the 6-month complete remission rate. CR, complete remission; TLS, tumor lysis syndrome; AKI, acute kidney injury. (DOC) [file pone.0055870.s001.doc]

**Table S1. Influence of the cause of acute kidney injury on the 6-month complete remission rate**

|  | **n (%)** | **% of CR** | **Odds ratio** | **95% confidence interval** | ***P* value** |
| --- | --- | --- | --- | --- | --- |
| **No AKI** | 54 (27%) | 40 (74) | 1.00 |  |  |
| **Cause of AKI** |  |  |  |  | <0.01 |
| TLS only | 29 (14%) | 20 (69) | 0.78 | [0.28;2.14] |  |
| TLS and other cause(s) | 34 (17%) | 11 (32) | 0.17 | [0.06;0.44] |  |
| Other cause(s) than TLS | 82 (41%) | 26 (32) | 0.16 | [0.07;0.36] |  |

CR, complete remission; TLS, tumor lysis syndrome; AKI, acute kidney injury
