# Supplementary material for: Acute Kidney Injury in Patients with Newly Diagnosed High-Grade Hematological Malignancies: Impact on Remission and Survival
Source: PLoS One. 2013 Feb 14;8(2):e55870. doi: 10.1371/journal.pone.0055870 (PMC3573047; doi:10.1371/journal.pone.0055870)
Supplement: Table S2 — Multivariate analysis: predictors of AKI. (DOC) [file pone.0055870.s002.doc]

**Table S2: Multivariate analysis: predictors of AKI**

| **Variables** | **Odds Ratio** | **95% confidence interval** | ***P* value** |
| --- | --- | --- | --- |
| Time (days) from hospital to ICU admission | 0.99 | [0.98;1.01] | 0.36 |
| Uric acid /10 µmol/L | 1.04 | [1.02;1.06] | <0.01 |
| Lactate dehydrogenase/1000 Units/L | 1.03 | [1;1.05] | 0.04 |
| Leukocyte count (G/L)  [1.0 – 30.0] | 1 |  |  |
| > 30.0 | 2.56 | [1.18;5.54] | 0.01 |
| < 1.0 | 1.38 | [0.52;3.67] | 0.52 |
|  |  |  | 0.04 |
| Disseminated intravascular coagulopathy | 1.88 | [0.82;4.34] | 0.14 |
